# International practice variation in perioperative laboratory testing in glioblastoma patients—a retrospective cohort study

**DOI:** 10.1007/s00701-021-05090-w

**Published:** 2022-01-07

**Authors:** Joeky T. Senders, Sybren L. N. Maas, Kaspar Draaisma, John J. McNulty, Joanna L. Ashby, Imo Hofer, Wouter W. van Solinge, Maarten ten Berg, Tom J. Snijders, Tatjana Seute, Pierre A. Robe, William B. Gormley, Timothy R. Smith, Marike L. D. Broekman

**Affiliations:** 1 Department of Neurosurgery, University Medical Center Utrecht, Utrecht University, Heidelberglaan 100, 3584 CX Utrecht, The Netherlands; 2 Department of Pathology, University Medical Center Utrecht, Utrecht University, Heidelberglaan 100, 3584 CX Utrecht, The Netherlands; 3 Computational Neuroscience Outcomes Center, Department of Neurosurgery, Brigham and Women’s Hospital, Harvard Medical School, 75 Francis St, Boston, MA 02115 USA; 4 Laboratory for Clinical Chemistry and Hematology, University Medical Center Utrecht, Utrecht University, Heidelberglaan 100, 3584 CX Utrecht, The Netherlands; 5 Department of Neurology, University Medical Center Utrecht, Utrecht University, Heidelberglaan 100, 3584 CX Utrecht, The Netherlands; 6 Department of Neurosurgery, Haaglanden Medical Center and Leiden University Medical Center, Lijnbaan 32, 2512 VA The Hague, The Netherlands

**Keywords:** Brain tumor, Glioblastoma, Laboratory testing, Practice variation

## Abstract

**Purpose:**

Although standard-of-care has been defined for the treatment of glioblastoma patients, substantial practice variation exists in the day-to-day clinical management. This study aims to compare the use of laboratory tests in the perioperative care of glioblastoma patients between two tertiary academic centers—Brigham and Women’s Hospital (BWH), Boston, USA, and University Medical Center Utrecht (UMCU), Utrecht, the Netherlands.

**Methods:**

All glioblastoma patients treated according to standard-of-care between 2005 and 2013 were included. We compared the number of blood drawings and laboratory tests performed during the 70-day perioperative period using a Poisson regression model, as well as the estimated laboratory costs per patient. Additionally, we compared the likelihood of an abnormal test result using a generalized linear mixed effects model.

**Results:**

After correction for age, sex, IDH1 status, postoperative KPS score, length of stay, and survival status, the number of blood drawings and laboratory tests during the perioperative period were 3.7-fold (*p* < 0.001) and 4.7-fold (*p* < 0.001) higher, respectively, in BWH compared to UMCU patients. The estimated median laboratory costs per patient were 82 euros in UMCU and 256 euros in BWH. Furthermore, the likelihood of an abnormal test result was lower in BWH (odds ratio [OR] 0.75, *p* < 0.001), except when the prior test result was abnormal as well (OR 2.09, *p* < 0.001).

**Conclusions:**

Our results suggest a substantially lower clinical threshold for ordering laboratory tests in BWH compared to UMCU. Further investigating the clinical consequences of laboratory testing could identify over and underuse, decrease healthcare costs, and reduce unnecessary discomfort that patients are exposed to.

**Supplementary Information:**

The online version contains supplementary material available at 10.1007/s00701-021-05090-w.

## Introduction

### Background

Although standard-of-care has been defined for the treatment of glioblastoma patients [11], substantial practice variation continues to exist in the day-to-day clinical management, particularly with regard to the ordering of diagnostic tests. These differences can reflect significant over—and underuse of diagnostic tests during the perioperative period, which in turn drive healthcare costs and expose patients to unnecessary discomfort. Furthermore, the use of diagnostic tests during the perioperative period provides an impression of the intensity of care delivered in the treatment of glioblastoma patients.

### Objectives

The aim of this study was to investigate the international practice variation by comparing laboratory testing policies during the perioperative period of glioblastoma patients between two hospitals—one in Boston, MA, and the other in Utrecht, The Netherlands. Additionally, we compared the financial costs associated with these laboratory testing policies, as well as the a priori likelihood of an abnormal test result to gain insight into the clinical threshold for ordering laboratory tests.

## Materials and methods

This study was conducted and reported according to the STrengthening the Reporting of OBservational studies in Epidemiology (STROBE) Statement. The Institutional Review Boards of University Medical Center Utrecht (UMCU) and Brigham and Women’s Hospital (BWH) approved this study and waived the need for informed consent because of its retrospective, observational study design.

### Study design, setting, and participants

In this retrospective cohort study, data from UMCU in Utrecht, The Netherlands, and BWH in Boston, USA, were used. Both are academic and tertiary referral centers for neurosurgical care. All adult patients who underwent craniotomy for a histologically confirmed glioblastoma between the 1st of January 2005 and 31st of December 2013, were included. To enhance the comparability between the two patient populations, we excluded patients that did not receive standard-of-care defined as maximal safe resection followed by chemoradiation.

### Outcomes and covariates

The outcome measures utilized in this study included (1) the total number of blood collections between 35 days before and 35 days after surgery; (2) the number and type of laboratory tests; (3) the a priori likelihood of an abnormal test result; and (4) the total cost price per patient during the perioperative period. To specify, a venipuncture is considered to be a single blood collection on which multiple laboratory test could be performed. The predictor of interest was the institution at which the patient was treated. Age in years, sex, isocitrate dehydrogenase 1 (IDH1) mutation status, postoperative Karnofsky Performance Scale (KPS) (< 70 versus ≥ 70), length of stay in days, survival status (< 1 year versus ≥ 1 year), timing of the laboratory test (day of surgery versus any other day), and value of the previous, similar laboratory test (no previous/normal test versus abnormal test) were collected as covariates. Missing data was multiply imputed by means of a random forest algorithm [14].

### Statistical analysis

Differences in baseline characteristics between patients treated at UMCU and BWH were tested by means of the Fisher’s exact test, independent-samples *t*-test, or Mann–Whitney *U* test, dependent on the variable type (i.e., categorical, count, or continuous) and distribution in case of numeric variables (i.e., normal or non-normal). The mean and standard deviation were used for normally distributed data, and the median and interquartile range (IQR) were used for non-normally distributed continuous data or count data. Normality was assessed graphically and tested by means of the Shapiro–Wilk test. Descriptive differences in the total number of blood collections (combined and throughout the 10-day perioperative period), laboratory tests (combined and stratified for the ten most frequently ordered laboratory tests), and total cost price per patient were assessed graphically and numerically by the median and IQR. To enhance the comparability of these estimates, we utilized the prices of a single institution (UMCU) for both institutions. The total cost price per patient included the price of the individual laboratory studies, as well as the order rate of distinct blood collections.

The multivariable analysis included two patient-level (i.e., one patient is one observation) and one laboratory test-level analysis (i.e., one laboratory test is one observation). The two patient-level analyses compared the differences in the total number of blood collections and the total number of distinct laboratory tests in patients treated at UMCU and BWH, after correcting for age, sex, IDH1 status, postoperative KPS score, length of stay, and survival status. Because these outcomes constitute discrete count data, a Poisson regression model was used. The laboratory test-level analysis compared the a priori likelihood of an abnormal laboratory finding between UMCU and BWH. In addition to the previously mentioned covariates, we corrected for the timing of the laboratory test and the value of the prior laboratory study. We included an interaction term to examine whether the prior laboratory value serves as an effect modifier. Lastly, to account for the correlation of laboratory studies of the same type or performed in the same patient, we used a multi-level generalized linear mixed effects model including individual laboratory tests and patients as random effects in a hierarchical fashion. *P*-values were adjusted for multiple testing by means of the Bonferroni correction based on 26 comparisons. A *p*-value below 0.05 was considered to be statistically significant. All statistical analyses were performed in R (Foundation for Statistical Computing, Vienna, Austria version 3.5.1) [9].

## Results

### Participants

In total, 969 patients underwent craniotomy for a histologically confirmed glioblastoma between January 2005 and December 2013 at one of the institutions (417 at UMCU and 552 at BWH). Of these patients, 230 (23.7%) were excluded because they were not treated by means of maximal safe resection followed by chemoradiation (UMCU 151 patients, 36.2%; BWH 79 patients, 14.3%). Consequently, a total of 739 patients were included in the final analysis (UMCU 266 patients, 36.0%; BWH 473 patients, 64.0%). Missing data were multiply imputed for IDH1 mutation status (46.0%), postoperative KPS score (30.1%), and survival status (7.3%). Baseline characteristics for all study participants (mean age 59.9 ± 12.1 years, 60.6% males) compared by institution are shown in Table 1. Compared to BWH, patients treated at UMCU were younger at time of surgery and had better postoperative KPS scores, longer length of stay, and better survival status. The distribution of all variables in the total cohort is depicted in Supplementary Figure S1.

**Table 1:** Baseline characteristics of the total cohort compared by institution

Patient characteristics	Level	UMCU (*n* = 266)		BWH (*n* = 473)		*p*
		*n*	%	*n*	%	
Age	< 50	61	22.9	72	15.2	** < 0.001**
	50–70	176	66.2	294	62.2	
	> 70	29	10.9	107	22.6	
	Mean ± SD	57.31 ± 11.58		61.39 ± 12.14		** < 0.001**
Sex	Male	163	61.3	285	60.3	0.845
	Female	103	38.7	188	39.7	
IDH1	Wildtype	246	92.5	434	91.8	0.695
	Mutant	20	7.5	39	8.2	
Post-op KPS score	≥ 70	235	88.3	380	80.3	**0.007**
	< 70	31	11.7	93	19.7	
Length of stay	Median [IQR]	7 [5–8]		4 [3–6]		** < 0.001**
Survival	≥ 1 year	190	71.4	279	59.0	**0.001**
	< 1 year	76	28.6	194	41.0	

Abbreviations: *BWH*, Brigham and Women’s Hospital; *IDH1*, isocitrate dehydrogenase; *IQR*, interquartile range; *KPS*, Karnofsky performance scale; *n*, sample size; *p*, p-value; *post-op*, postoperative; *SD*, standard deviation; *UMCU*, University Medical Center Utrecht

The bold value represents the significant differences in baseline characteristics between the UMCU and BWH cohort

### Descriptive outcomes

**Fig. 1:**
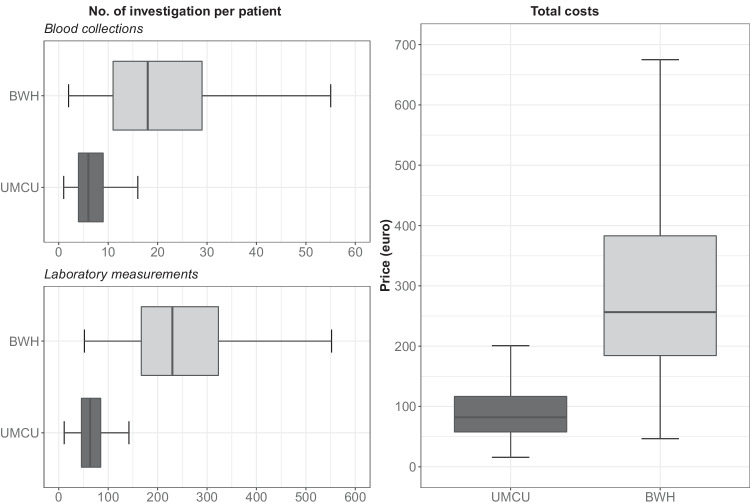
Box plots depicting the median number of blood collections and laboratory measurements ordered during the 70-day perioperative period compared by institution (left), as well as the associated costs per patient (right). The middle line represents the median, the boxes the 50% confidence interval (i.e., the interquartile range), and the whiskers the 95% confidence interval

**Fig. 2:**
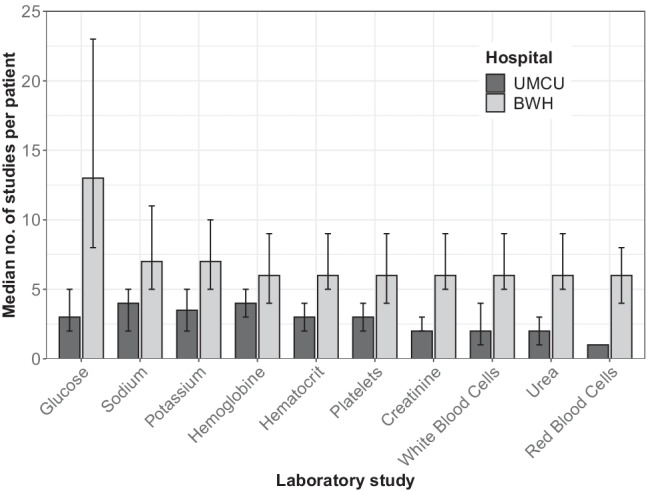
Bar chart depicting median number of laboratory studies during the 70-day perioperative period of the ten most frequently ordered test compared by institution. The error bars reflect the 50% confidence interval (i.e., interquartile range)

**Fig. 3:**
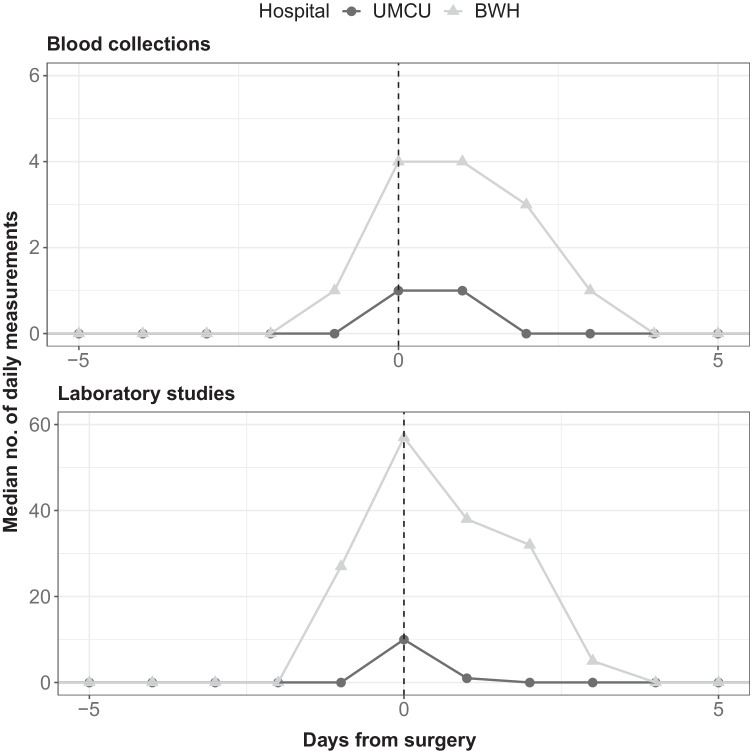
Line plot depicting the mean number of distinct blood collections and laboratory studies per day throughout the 20-day perioperative period

The median number of distinct blood collections during the 70-day perioperative period was 6 (IQR 4–9) in UMCU and 18 (IQR 11–29) in BWH (Fig. 1). The median number of laboratory tests during the 70-day perioperative period was 64 (IQR 46–85) in UMCU and 230 (IQR 167–323) in BWH. Differences in the frequency of ordering laboratory studies were observed across the spectrum of laboratory tests, yet most pronounced with regard to the testing of glucose levels (Fig. 2). Differences in the rate of laboratory testing were most evident on the day of surgery and the day after surgery (Fig. 3). Financially, the median cost price for laboratory testing during the perioperative period was 82 euros per patient (IQR 58–117) in UMCU and 256 euros per patient (IQR 184–383) in BWH (Fig. 1).

### Statistical analysis

The number of blood samples drawn from glioblastoma patients operated at BWH was estimated to be 3.7-fold higher compared to patients from UMCU after correction for age, sex, IDH1 mutation status, postoperative KPS score, length of stay, and survival status (Table 2). Older age, female sex, IDH1 mutation, postoperative KPS scores lower than 70, longer length of stay, and short survival status were all positively associated with the number of blood collections performed during the perioperative period (Table 3). The number of distinct laboratory tests performed on glioblastoma patients operated at BWH was estimated to be 4.7-fold higher compared to patients from UMCU after correcting for age, sex, IDH1 status, postoperative KPS score, length of stay, and survival. Older age, female sex, IDH1 wildtype, postoperative KPS score lower than 70, longer length of stay, and shorter survival status were also positively associated with the number of laboratory tests performed during the perioperative period.

**Table 2:** Poisson regression model into the number of blood collections performed during the perioperative period

Patient characteristic	Level	Count ratio	95% CI	p*
Age	< 50 years	Ref	Ref	Ref
	50–70 years	1.11	1.02–1.21	0.0052
	> 70 years	1.14	1.03–1.26	0.0026
Sex	Male	Ref	Ref	Ref
	Female	1.06	1.00–1.12	0.0312
IDH1	Wildtype	Ref	Ref	Ref
	Mutant	1.12	1.00–1.26	0.0624
Post-op KPS score	≥ 70	Ref	Ref	Ref
	< 70	1.50	1.41–1.60	< 0.001
Length of stay	Per day increase	1.06	1.06–1.07	< 0.001
Survival	≥ 1 year	Ref	Ref	Ref
	< 1 year	1.23	1.16–1.30	< 0.001
Hospital	UMCU	Ref	Ref	Ref
	BWH	3.66	3.38–3.96	** < 0.001**

Abbreviations: *BWH*, Brigham and Women’s Hospital; *IDH1*, isocitrate dehydrogenase; *KPS*, Karnofsky performance scale; *p*, p-value; *post-op*, postoperative; *UMCU*, University Medical Center Utrecht

The bold values represents statistically significant differences in the use of perioperative laboratory testing between the two institutions

^*^Adjusted for multiple testing by means of a Bonferroni correction based on 26 comparisons

**Table 3:** Poisson regression model into the number of laboratory studies performed during the perioperative period

Patient characteristic	Level	Count ratio	95% CI	p*
Age	< 50 years	Ref	Ref	Ref
	50–70 years	1.02	1.00–1.05	0.14
	> 70 years	0.96	0.93–0.99	< 0.001
Sex	Male	Ref	Ref	Ref
	Female	1.02	1.00–1.04	0.0104
IDH1	Wildtype	Ref	Ref	Ref
	Mutant	0.91	0.88–0.95	< 0.001
Post-op KPS score	≥ 70	Ref	Ref	Ref
	< 70	1.34	1.31–1.36	< 0.001
Length of stay	Per day increase	1.06	1.06–1.06	< 0.001
Survival	≥ 1 year	Ref	Ref	Ref
	< 1 year	1.07	1.05–1.08	< 0.001
Hospital	UMCU	Ref	Ref	Ref
	BWH	4.65	4.53–4.77	** < 0.001**

Abbreviations: *BWH*, Brigham and Women’s Hospital; *IDH1*, isocitrate dehydrogenase; *KPS*, Karnofsky performance scale; *p*, p-value; *post-op*, postoperative; *UMCU*, University Medical Center Utrecht

The bold values represents statistically significant differences in the use of perioperative laboratory testing between the two institutions

^*^Adjusted for multiple testing by means of a Bonferroni correction based on 26 comparisons

The a priori likelihood of an abnormal test result was lower in BWH compared to UMCU (odds ratio [OR] 0.75, 95% CI 0.67–0.85), after correction for age, sex, IDH1 mutation status, postoperative KPS score, survival status, timing of the laboratory test, and value of the prior laboratory test (Table 4). However, whenever the prior lab finding was abnormal, the likelihood of an abnormal finding in BWH patients was higher compared to UMCU (OR 2.09, 95% CI 1.73–2.52). Laboratory studies performed in older patients, in patients with a KPS score lower than 70 or with a short survival status, performed on the day of surgery, or preceded by an abnormal finding were more likely to be abnormal as well.

**Table 4:** Multi-level generalized linear mixed effects model into the likelihood of an abnormal laboratory finding. This is a laboratory study-level analysis; therefore, one observation represents a single laboratory measurement. In this multi-level model, random effects were specified to account for the correlation among laboratory studies of the same type and the same patient in a hierarchical fashion

Characteristic	Level	OR	95% CI	p*
Age	< 50	Ref	Ref	Ref
	50–70	1.16	1.01–1.33	0.0154
	> 70	1.19	1.01–1.39	0.0289
Sex	Male	Ref	Ref	Ref
	Female	0.96	0.88–1.05	> 0.99
IDH1	Wildtype	Ref	Ref	Ref
	Mutant	1.13	0.94–1.36	> 0.99
Post-op KPS score	≥ 70	Ref	Ref	Ref
	< 70	1.30	1.16–1.45	< 0.001
Survival	≥ 1 year	Ref	Ref	Ref
	< 1 year	1.18	1.07–1.29	< 0.001
Study on day of surgery	No	Ref	Ref	Ref
	Yes	2.12	1.99–2.26	< 0.001
Prior lab finding	Normal	Ref	Ref	Ref
	Abnormal	2.92	2.43–3.50	< 0.001
Hospital	UMCU	Ref	Ref	Ref
	BWH	0.75	0.67–0.85	** < 0.001**
Interactions				
Hospital and prior lab value	BWH and abnormal	2.09	1.73–2.52	** < 0.001**

Abbreviations: *BWH*, Brigham and Women’s Hospital; *IDH1*, isocitrate dehydrogenase; *KPS*, Karnofsky performance scale; *p*, p-value; *post-op*, postoperative; *UMCU*, University Medical Center Utrecht

The bold values represents statistically significant differences in the use of perioperative laboratory testing between the two institutions

^*^Adjusted for multiple testing by means of a Bonferroni correction based on 26 comparisons

## Discussion

In glioblastoma patients, the number of blood collections and laboratory tests performed during the perioperative period is substantially higher (3.7- and 4.7-fold, respectively) in BWH compared to UMCU. This was associated with a similar substantial increase in laboratory costs. Furthermore, the a priori likelihood of an abnormal test result was lower in the US cohort, except when the prior finding was abnormal. In this case, the likelihood of an abnormal finding was higher.

### Implications

Our results suggest substantial practice variation regarding laboratory testing policies in glioblastoma patients during the perioperative period and imply a lower clinical threshold for ordering laboratory tests in the US institution. The lower clinical threshold encompasses both the ordering of novel laboratory tests and the follow-up of abnormal laboratory finding.

Similar findings were reported in a previous study on international practice variation demonstrating a significant difference in the number of postoperative CT scans ordered after burr hole drainage for a chronic subdural hematoma (median of 0 scans in the Dutch institution and 4 in the US institution) [3]. Furthermore, this study found that all re-interventions were preceded by clinical decline, suggesting little benefit of routine scanning in asymptomatic patients.

The results of the current study should be interpreted with caution, especially when generalizing them to practice variation between the two countries or even the two continents. Namely, several studies have already demonstrated significant practice variation in the treatment of patients with traumatic brain injury between countries within the same continent [6, 12, 13]. Furthermore, two recent studies also found substantial differences in the use of laboratory tests in the primary care setting between regions within the same country [7, 8]. This group also emphasizes the importance of investigating practice variation in laboratory testing policies as it constitutes one of the most variable cost items in healthcare due to its frequent and inconsistent use. De Witt Hamer et al. found significant variation in glioblastoma overall survival trends between hospitals in the Netherlands. However, this variation was associated with patient-related factors (e.g., age and functional status) rather than hospital-related factors (e.g., academic setting or case volume) [2].

It should be underlined that the current study has only demonstrated and quantified differences in the use of laboratory tests between a US and Dutch institution. Although no data on the clinical and therapeutic consequences of laboratory test are available, the striking difference in testing policies implies substantial over- or underuse of laboratory tests in one of the included institutions. Further investigating the clinical implications can identify potential over- and underuse of laboratory tests. According to a 2017 survey by Lyu et al., US physicians estimate that as much as 24.9% of all medical tests ordered are unnecessary [5]. While this survey is subjective in nature and offers no quantifiable evidence, it provides a valuable estimate of the magnitude of medical overuse. Even slight improvements in the use of laboratory tests could already have a significant impact on healthcare costs.

Furthermore, it remains to be elucidated what underlying mechanisms drive these differences in practice variation [10]. Defensive medicine has been formulated as one of the underlying drivers and refers to physicians altering their clinical behavior because of the threat of malpractice liability. Two other reported drivers are disparities in technology and expertise, as well as differences in the healthcare reimbursement model [1, 4]. The magnitude of these potential drives and the role of other potential drivers of practice variation remain to be explored as well.

### Limitations

A few limitations should be mentioned. First, this study only compared data from two institutions. In order to validate and improve the generalizability of these results, inclusion of data from more institutions would be desirable. Second, differences were observed in participant selection. BWH were more likely to receive maximal safe resection despite older age and poorer functional status, which resulted in a cohort with less favorable characteristics compared to the UMCU cohort. Although we aimed to reduce the effect of selection bias by including all potential confounders in the analysis, residual confounding of covariates that have not been measured or documented could still influence the results. Third, on average 6.4% of all data points were missing in the total data set, which was multiply imputed by means of a random forest algorithm to mitigate the risk of systematic bias associated with a complete-case analysis. Missingness was confined to covariates included as confounders. No missingness was observed among the outcomes or the exposure of interest. Fourth, laboratory tests performed at different institutions during the 35-day perioperative period were not included in the analysis. As such, the current findings resemble the differences on institutional level, which provides a mere indication of the differences on regional and national level. Lastly, to enhance the comparability of the cost analysis, we utilized the same laboratory cost price for both centers. However, the actual onsite cost prices of the ten most frequently ordered laboratory tests, as depicted in Fig. 2, were 30 to 80 times higher in BWH. This means that the current study reflects the minimal difference in cost price per patient, but that the actual difference is even more substantial. Furthermore, the financial analysis only reflects the direct primary laboratory costs, but not the consequential secondary costs (e.g., delaying discharge, additional diagnostics and treatments) or patient harms and discomfort. Despite these limitations, we believe the current study provides valuable insights into the international practice variation in perioperative laboratory testing policies in glioblastoma patients.

Future studies should further investigate the practice variation in laboratory testing, as well as other diagnostic and therapeutic modalities. Comparing practice variation between regions with different legal environments can provide insight into the potential role of defensive medicine. Other potential drivers of practice remain to be elucidated as well. Evaluating the clinical impact of distinct diagnostic tests, both negative and positive, could facilitate harmonization and effective usage of healthcare resources. Even slight optimization in laboratory testing policies could make substantial impact on the increasing monetary burden of healthcare and reduce unnecessary discomfort patients are exposed to.

## Conclusion

Our results suggest substantial practice variation with regard to laboratory testing policies during the perioperative period of glioblastoma patients and imply a lower clinical threshold for ordering laboratory tests in BWH. Further investigating the clinical consequences of laboratory tests could potentially identify overuse and underuse, decrease healthcare costs, and reduce unnecessary discomfort patients are exposed to.

## Supplementary Information

Below is the link to the electronic supplementary material.

Supplementary file1 (DOCX 39 KB)

## Data Availability

The laboratory and clinical information used in the current study contain sensitive patient information. Therefore, the Health Insurance Portability and Accountability Act (HIPAA) prohibits public distribution of the original reports.

## Code availability

This current study does not utilize any custom computer code or software. The experiments performed in this study are reproducible after following the steps outlined in the methods section. The written computer code is available from one of the authors (Joeky Senders: j.t.senders@umcutrecht.nl) on any reasonable request.
