# Supplementary material for: International practice variation in perioperative laboratory testing in glioblastoma patients—a retrospective cohort study
Source: Acta Neurochir (Wien). 2022 Jan 7;164(2):385–92. doi: 10.1007/s00701-021-05090-w (PMC8854260; doi:10.1007/s00701-021-05090-w)
Supplement: Supplementary file 1 — Supplementary file1 (DOCX 39 KB) [file 701_2021_5090_MOESM1_ESM.docx]

**Supplementary Figure S1.** Variable distribution in the total study cohort.
